# Depletion of *GPSM1* enhances ovarian granulosa cell apoptosis via cAMP-PKA-CREB pathway in vitro

**DOI:** 10.1186/s13048-020-00740-6

**Published:** 2020-11-21

**Authors:** Xuzi Cai, Huijiao Fu, Yan Wang, Qiwen Liu, Xuefeng Wang

**Affiliations:** Department of Obstetrics and Gynecology, The Third Affiliated Hospital of Southern Medical University, No. 183 West Zhongshan Avenue, Guangzhou, 510000 Guangdong China

**Keywords:** Premature ovarian insufficiency, Whole-exome sequencing, *GPSM1*, Ovarian granulosa cell, cAMP-PKA-CREB pathway

## Abstract

**Background:**

Genetic causes of premature ovarian insufficiency (POI) account for approximately 20 ~ 25% of patients. So far, only a few genes have been identified.

**Results:**

Here, we first identified the c.1840C > A on G-protein signaling modulator 1 (*GPSM1*) as a susceptibility locus for POI in 10 sporadic POI patients by whole-exome sequencing. The frequency of *GPSM1* c.1840C > A was then verified as 3/20 in a POI sample of 20 patients (including the above 10 patients) by Sanger sequencing. RT-PCR and western blot analysis showed the expression of GPSM1 in rat ovaries was increased in the large antral follicle stage compared to the primordial follicle stage (*P* < 0.01). The cell proliferation assay (CCK8) and flow cytometry suggested that the small-interfering RNA-induced silencing of *Gpsm1* significantly increased apoptosis and decreased proliferation of rat ovarian granulosa cells (GCs) (*P* < 0.01). Furthermore, suppression of *Gpsm1* in GCs reduced levels of cAMP, PKAc, p-CREB as well as the ratio of Bcl-2/Bax, and increased the expression of Caspase-3 and Cleaved Caspase-3 (*P* < 0.01).

**Conclusions:**

In summary, this study identified a susceptibility variant *GPSM1* c.1840C > A of POI for the first time. *Gpsm1* was related to rat follicle development, and silencing of *Gpsm1* increased apoptosis and decreased proliferation in rat GCs, possibly through inhibition of the cAMP-PKA-CREB pathway.

**Supplementary Information:**

The online version contains supplementary material available at 10.1186/s13048-020-00740-6.

## Introduction

Premature ovarian insufficiency (POI) is a clinical syndrome characterized by the loss of ovarian activity before the age of 40 years, which results in hypergonadotropic hypogonadism [1]. It affects approximately 1% women under 40 years old [2]. The opportunity to preserve fertility in women with POI is poor, which has a negative impact on both the psychological and reproductive health of women of childbearing age. As women are marrying at a later age, infertility caused by POI is becoming a greater concern. Early detection of the high-risk population would allow for increased treatment options, and perhaps the POI-related infertility could be avoided. Research into the pathogenesis of POI is particularly important for molecular diagnosis and prevention.

Although most POI cases are idiopathic, genetic factors are considered to be one of the main causes of POI [3], accounting for 20–25% [4]. Currently, only several genes have been confirmed to be associated with the pathogenesis of POI, including *FOXL2*, *BMP2*, *NOBOX*, *FIGLA*, and *GDF9* [5]. The screening and validation of POI candidate genes is a large task. Currently, most of the research on POI-pathogenesis genes is focused on pedigree studies [6, 7]. However, POI presents with high genetic heterogeneity; each family or individual patient seems to be unique in the pathogenesis and there is no significant definitive pathogenic gene for POI. If common pathogenic mutations can be sought out in sporadic POI populations, more insight into the genetic changes of POI patients can be gained. In recent years, with the development and maturity of high-throughput sequencing technologies, whole-exome sequencing (WES) has been widely applied to explore new pathogenic genes of hereditary disease, and has been verified as an effective tool for research into genetic etiology [8–10].

In this study, we used WES to initially identify a POI susceptibility gene in unrelated Chinese women affected by POI. A functional study in vitro was then carried out using rat ovarian granulosa cells (GCs). The outcome of this study provided an insight of the etiology of POI by providing new candidates and pathways.

## Results

### WES and sanger sequencing identified heterozygous *GPSM1* mutation in 3 POI patients

**Fig. 1:**
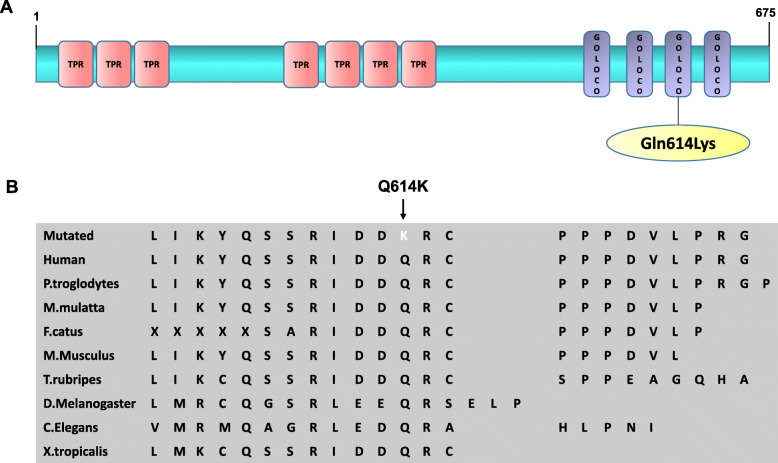
Hotspot and conservation sites of GPSM1 Q614K. **a**. Functional domain information of Glu614Lys (Q614K) in protein GPSM1. **b**. Conservation analysis of GPSM1 Q614K among different species

We performed WES on 10 POI patients. The mean coverage of the target region is 99.8%. More than 98.76% of the target was covered at 20× depth. We applied the following exome filtration procedure: total variants called in 10 patients and variants that are absent or with minor allele frequency less than 1% in the population public databases (dbSNP, 1000Genomes, and ExAC). After these filters, a total of 12 genes including 22 variants were retained: *OR2T29 (*c.26A > G), *ANKRD36C* (c.1265 T > G), *FRG1* (c.330G > T), *PSPH* (c.268G > A), *PABPC3* (c.541G > A, c.691A > G, c.832C > T, c.859A > G, c.938C > T, c.956C > T), *LMO7* (c.911G > A), *TPSAB1* (c.422C > T), *TBC1D26* (c.167A > C), *CNN2* (c.629 T > C, c.630G > A, c.632G > T, c.670G > A, c.680G > A, c.695C > A), *DKKL1* (c.71 T > G), *SCUBE1* (c.1169C > G), and *GPSM1* (c.1840C > A). All the above mutations presented as heterozygous. Details of all variants are listed in Table 1. Of these candidate genes, *GPSM1* (G-protein signaling modulator 1) sparked our interest. For *GPSM1* (c.1840C > A), the ExonicFunc. refGene, rs number, mutation frequency in ExAC, mutation frequency in 1000G(all), SIFT score, Ployphen2 score were missense mutation, rs539775258, 0.000008446, 0.0002, 0.001, and 0.998, respectively. Protein alignments revealed the GPSM1 p. Glu614Lys variant, located in the third G-protein regulatory (GPR) motif of the protein, affected an amino acid highly conserved among species (Fig. 1). Sanger sequencing confirmed that *GPSM1* (c.1840C > A) were heterozygous in 3/20 patients.

**Table 1:** Information about candidate gene (ND: no date)

Gene	Chr	Transcription ID	exon	Nucleotide change	AA change	dbSNP	Frequency	Pathogenic predictions						Genotype	Number of variant samples
							1000 genomes	ExAC	Ployphen2	SIFT	Mutation Taster	LRT	Mutation Assessor		
OR2T29	Chr1	NM_001004694	1	c.26A > G	p.N9S	ND	ND	0.00009122	Benign	Deleterious	Probably harmless	Neutral	Medium	Heterozygous	5
ANKRD36C	Chr2	NM_001310154	16	c.1265 T > G	p. V422G	rs78715705	ND	0.0001	ND	Deleterious	Probably harmless	ND	ND	Heterozygous	4
FRG1	Chr4	NM_004477	5	c.330G > T	p. K110N	ND	ND	0.009	Probably damaging	Deleterious	Disease causing	Deleterious	Medium	Heterozygous	5
sPSPH	Chr7	NM_004577	5	c.268G > A	p. G90S	rs75395437	0.0002	0.0051	Probably damaging	Deleterious	Disease causing	Deleterious	Medium	Heterozygous	9
PABPC3	Chr13	NM_030979	1	c.541G > A	p. A181T	rs112107735	0.007189	0.0013	Possible	Deleterious	Disease causing	Unknown	Medium	Heterozygous	8
PABPC3	Chr13	NM_030980	1	c.691A > G	p. K231E	rs78826513	ND	0.002	Probably damaging	Deleterious	Disease causing	Unknown	Medium	Heterozygous	9
PABPC3	Chr13	NM_030981	1	c.832C > T	p. R278C	rs78552667	ND	0.0001	Benign	Deleterious	Disease causing	Unknown	Medium	Heterozygous	9
PABPC3	Chr13	NM_030982	1	c.859A > G	p. R287G	rs201411821	ND	0.0002	Benign	Deleterious	Disease causing	Unknown	High	Heterozygous	9
PABPC3	Chr13	NM_030983	1	c.938C > T	p. A313V	rs76994938	0.000399	0.00001648	Benign	Deleterious	Probably harmless	Unknown	Neutral	Heterozygous	8
PABPC3	Chr13	NM_030984	1	c.95C > T	p. T319I	rs80261016	ND	0	Possible	Deleterious	Disease causing	Unknown	Low	Heterozygous	9
LMO7	Chr13	NM_001306080	10	c.1610G > A	p. R537K	rs142687160	0.009185	0.0037	Benign	Deleterious	Disease causing	Deleterious	Neutral	Heterozygous	5
TPSAB1	Chr16	NM_003294	4	c.422C > T	p. T141I	rs1064781	ND	0.0068	Benign	Deleterious	Probably harmless	Neutral	Neutral	Heterozygous	8
TBC1D26	Chr17	NM_178571	5	c.167A > C	p. E56A	rs3852810	ND	0.0099	Possible	Deleterious	Probably harmless	Unknown	Medium	Heterozygous	4
CNN2	Chr19	NM_001303501	7	c.809 T > C	p.M270T	rs200177867	ND	0.00006856	Probably damaging	Deleterious	Disease causing	Unknown	Medium	Heterozygous	5
CNN2	Chr19	NM_001303501	7	c.810G > A	p.M270I	rs201532581	ND	0.00006856	Probably damaging	Deleterious	Disease causing	Unknown	Medium	Heterozygous	5
CNN2	Chr19	NM_001303501	7	c.812G > T	p. G271V	rs199741851	ND	0.00006855	Probably damaging	Deleterious	Disease causing	Unknown	Medium	Heterozygous	5
CNN2	Chr19	NM_001303501	7	c.850G > A	p. G284S	rs77830704	ND	0.0003	Probably damaging	Deleterious	Disease causing	Unknown	Medium	Heterozygous	5
CNN2	Chr19	NM_001303501	7	c.860G > A	p. R287Q	rs78386506	ND	0.0002	Possible	Deleterious	Disease causing	Unknown	Medium	Heterozygous	5
CNN2	Chr19	NM_001303501	7	c.875C > A	p. P292H	rs75676484	ND	0.00008565	Probably damaging	Deleterious	Disease causing	Deleterious	Medium	Heterozygous	5
DKKL1	Chr19	NM_001197301	2	c.72 T > G	p. L25R	rs2303757	0.004193	0.0021	Possible	Deleterious	Harmless	Neutral	Neutral	Heterozygous	5
SCUBE1	Chr22	NM_173050	10	c.1169C > G	p. P390R	rs185039637	0.001997	0.0011	Possible	Deleterious	Disease causing	Deleterious	Low	Heterozygous	3
GPSM1	Chr9	NM_001145638	14	c.1840C > A	p. Q614K	rs539775258	0.0002	0.000008446	Probably damaging	Deleterious	Disease causing	Deleterious	Medium	Heterozygous	3

### Increased level of GPSM1 in multi-follicle developed ovary

**Fig. 2:**
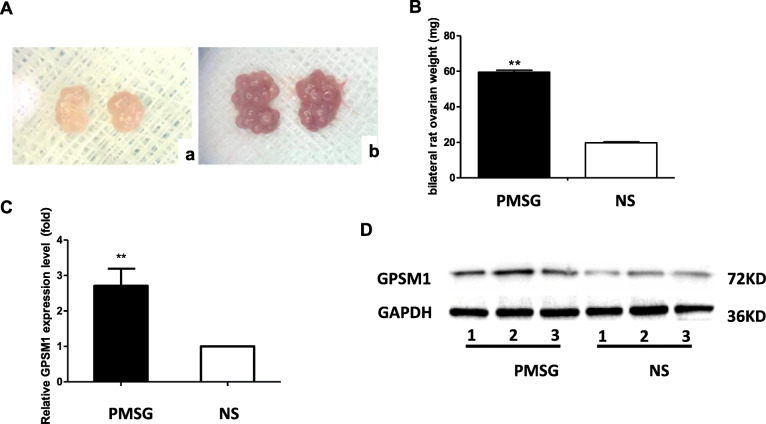
The expression of GPSM1 in ovaries before and after multi-follicle development. A. Morphological changes of rat ovaries: a. Ovaries from NS-treated immature rats were light pink and normal in size; b. Ovaries from PMSG-primed immature rats were significantly enlarged, with hyperemia and mulberry-like changes. B. The weight of the bilateral rat ovaries after treatment in the experimental group (PMSG group, *n* = 3) was significantly increased compared to the control group (NS group, *n* = 3) (^**^P < 0.01). C and D. The expression of GPSM1 detected by RT-PCR and western blot was up-regulated in the PMSG group (*n* = 3) compared to the NS group (*n* = 3) (^**^P < 0.01)

Multi-follicle development in immature rats was successfully promoted. The ovaries of the PMSG group were significantly enlarged, congestive, and multi-follicles developed compared to the NS group (Fig. 2a). The weight of the ovaries in the PMSG group increased significantly, about three times as much as the NS group (*P* < 0.01, Fig. 2b). A significant increase in GPSM1 mRNA and protein expression was observed in the PMSG group compared to the NS group, as demonstrated by RT-PCR and western blot analysis, respectively (*P* < 0.01, Fig. 2c, d).

### GPSM1 downregulated affects cell apoptosis and proliferation in rat GCs

**Fig. 3:**
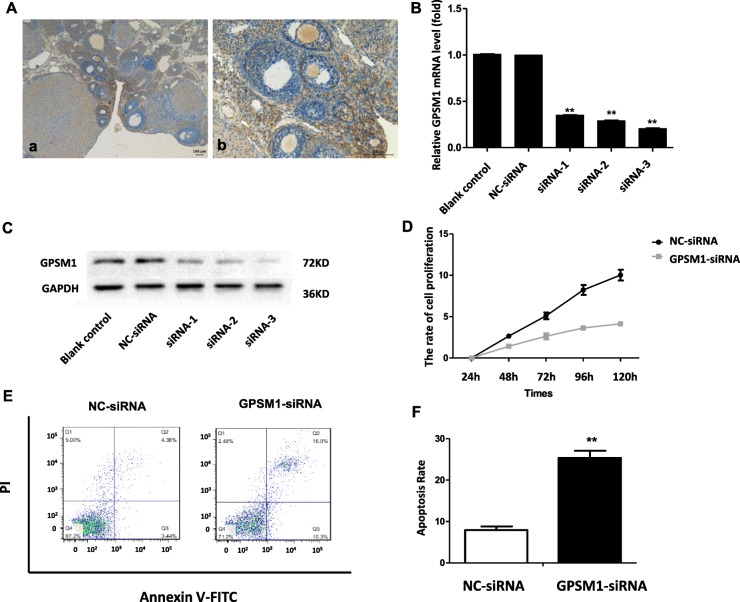
The effect of silencing GPSM1 on GCs. A. The expression of GPSM1 in rat ovaries detected by immunohistochemistry. B and C. Silencing of *Gpsm1* expression in GCs using siRNAs. RT-PCR (B) and western blot (C) analyses showed that *Gpsm1*-targeting siRNA-3 provided optimal depletion of GPSM1 in GCs compared to the siRNA-negative control (NC-siRNA) and blank control (^**^P < 0.01). D. *Gpsm1*-knockdown decreased the proliferation rate of GCs significantly 48 h, 72 h, 96 h, and 120 h after transfection with siRNA. GCs transfected with NC-siRNA or *Gpsm1*-siRNA were subjected to CCK8 analysis (^**^P < 0.01). E and F. *Gpsm1*-knockdown increased the apoptosis rate of GCs. GCs transfected with NC-siRNA or *Gpsm1*-siRNA were subjected to Annexin V-FITC/PI double staining and flow cytometric analysis (^**^P < 0.01)

GPSM1 was widely expressed in the rat ovaries (Fig. 3a), such as in oocytes, GCs, luteal cells, and stromal cells. Considering the significant role of GCs in follicular development, we further investigated the role of GPSM1 in GCs. Three siRNAs targeting *Gpsm1* (siRNA-1, siRNA-2, and siRNA-3) and a siRNA for a negative control (NC-siRNA) were designed and individually transfected into GCs. The results show that NC-siRNA had no significant effect on the expression of GPSM1, and siRNA-3 had the highest knockdown efficiency (> 70%) at both mRNA and protein levels (Fig. 3b, c); siRNA-3 was used in the following experiments. The CCK8 assay showed GCs in both groups were in a proliferative trend, but *Gpsm1*-knockdown markedly decreased proliferation at 48 h, 72 h, 96 h, and 120 h (*P* < 0.01, Fig. 3d). Flow cytometry analysis revealed that *Gpsm1*-knockdown significantly increased the apoptosis rate of GCs (*P* < 0.01, Fig. 3e, f).

### Downregulating of Gpsm1 alters the expression of the cAMP-CREB-PKA signaling molecules in GCs

**Fig. 4:**
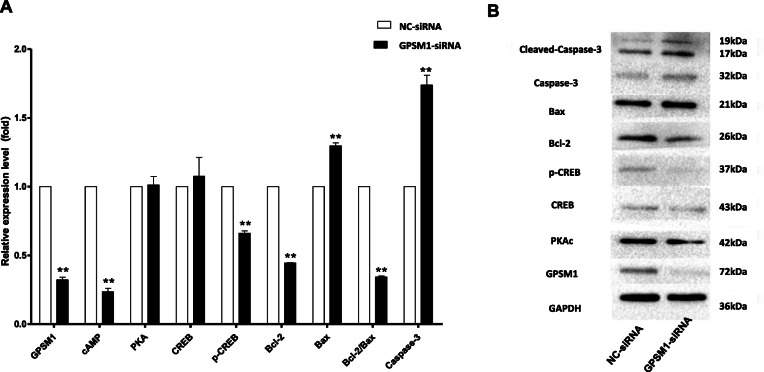
Silencing of *Gpsm1* alters the expression of the cAMP-CREB-PKA signaling molecules in GCs. After GCs were transfected with NC-siRNA and *Gpsm1*-siRNA, RT-PCR (**a**) and western blot (**b**) analyses were performed to measure the levels of GPSM1 and intracellular signaling molecules. The average results from three independent experiments are shown (^**^P < 0.01)

Next, we investigated the effect of *Gpsm1*-knockdown on intracellular signaling in rat GCs. RT-PCR revealed that the cAMP level decreased concomitantly with down-regulation of *Gpsm1* (*P* < 0.01, Fig. 4a). It is widely known the various effects of cAMP are achieved mainly through activation of cAMP-dependent protein kinase A (PKA). Unexpectedly, there was no significant difference in PKA mRNA expression between the two groups (*P* > 0.05, Fig. 4a). Therefore, we targeted PKAc, a catalytic subunit of PKA, protein levels for western blot analysis. The PKAc protein level was significantly reduced in the *Gpsm1*-silenced group compared to the NC group (*P* < 0.01, Fig. 4b). As some studies have reported that GPSM1 could regulate the phosphorylation of cAMP-response element binding protein (CREB) by mediating PKA [11], and p-CREB could regulate transcription of the apoptosis suppressor Bcl-2 [12], the CREB, p-CREB, and Bcl-2 protein levels were assessed. Both the RNA and protein expression of p-CREB and Bcl-2 were attenuated in the *Gpsm1*-silenced group compared to the NC group (*P* < 0.01, Fig. 4), while CREB expression showed no significant difference (*P* > 0.05, Fig. 4). To calculate the ratio of Bcl-2/Bax, Bax was quantified; the results showed an elevation of Bcl-2/Bax on RNA levels in the *Gpsm1*-silenced group (*P* < 0.01, Fig. 4a) but no difference between groups at the protein level (*P* > 0.05, Fig. 4b). However, the ratio of Bcl-2/Bax was noticeably decreased at both the RNA and protein levels. Finally, RNA and protein levels of Caspase-3, and protein levels of cleaved Caspase-3, were found to be elevated in the *Gpsm1*-silenced group (*P* < 0.01, Fig. 4).

## Discussion

From our study of 10 POI patients, we identified a possible POI susceptibility gene, *GPSM1*. The *GPSM1* gene was reported in public expression databases to be expressed in multiple types of tissue and cell, especially in ovary (https://www.ncbi.nlm.nih.gov/gene/26086). It encodes activator of G protein signaling 3 (AGS3), which was identified as an evolutionarily conserved protein [13, 14] and to be associated with cell division [15], cell proliferation [16], differentiation [17], autophagy [18] and so on. AGS3 has a modular domain structure consisting of seven tetratricopeptide repeats (TPRs) and four G-protein regulatory (GPR) motifs. It was a regulatory accessory protein of G-protein signaling which could bind preferentially to inactive Gα/o subunit complexed with guanine dinucleotide phosphate (GDP) at multiple GPR motif repeats [15, 19, 20]. Thus, it could regulate the production of cAMP which is one of the most important second messengers in pathways of metabolism, apoptosis, proliferation and material transport. The heterozygous variant of *GPSM1* c.1840C > A is a missense mutation occurring at the third GPR motif of ASG3, leading to a change in amino acid sequence, thus likely to be a highly pathogenic mutation. Sanger sequencing determined the frequency of *GPSM1* c.1840C > A in the small POI sample of 20 patients to be 3/20. This suggested *GPSM1* c.1840C > A might be involved in POI. To the best of our knowledge, this is the first report of *GPSM1* c.1840C > A in POI patients.

Early studies [21, 22] have shown that at 24 h following PMSG treatment, the ovaries of rats presented with multiple follicular growth, and at 48 h, the follicular antrum was extremely large and the parietal granulosa cell layer became very thin. Here, we first explored the expression pattern of GPSM1 at different stages of ovarian growth in rats. The results showed that the expression of GPSM1 was significantly increased in the large antral follicle stage compared to the primordial follicle stage. This result indicated that GPSM1 might play an important role in follicular growth in rats.

The apoptosis of GCs and follicular atresia have been shown to play key roles in the pathogenesis of POI [3, 23]. The mechanism by which GPSM1 regulates heterotrimeric G-proteins in GCs has yet to be elucidated. GPSM1, encoding AGS3, could compete with free G subunits for binding to Gα/o-GDP subunits, regulating downstream signal transduction pathways by inhibiting the Gi subunit. The Gi subunit directly inhibits adenylyl cyclase (AC) activity [24]. That is how GPSM1 conducts signals, by activating AC to promote the synthesis of cAMP. Changes in cAMP levels are a common observation in the growth and maturation of GCs [25, 26]. Thus, we initially postulated GPSM1 in GCs might play an important role in apoptosis by regulating the activation of cAMP pathways.

To verify this hypothesis, we first demonstrated the silencing of *Gpsm1* induced GC apoptosis and inhibited proliferation. Furthermore, it was confirmed that the generation of cAMP was correlated to the expression of GPSM1 in rat GCs. Next, we explored the downstream signaling molecules of cAMP in GCs. There was no decline in PKA levels, but PKAc decreased when GPSM1 was down-regulated, which suggested the subsequent signal transduction mediated by cAMP might not be transmitted by the change of PKA level, but probably by the level of PKAc, which is an active subunit decomposed from PKA. Concomitant with the reduction of PKAc, CREB, a target molecule of PKA, as well as an important mediator of multiple signal transduction pathways in GCs [27, 28], was found to be less phosphorylated in *Gpsm1*-knockdown cells. Bcl-2 is a well-known target of CREB [12], and was also reported as one of the important apoptosis suppressors in GCs [29]. Bax-dependent apoptosis is a common pathway of cell death and the balance of Bcl-2 and Bax is a key determinant of the survival or death of GCs. As expected, the level of Bcl-2 and the ratio of Bcl-2/Bax were found to be markedly reduced after down-regulation of GPSM1. Finally, as apoptosis markers, Caspase-3 and cleaved Capase-3 showed an uptrend in *Gpsm1*-knockdown cells.

## Conclusions

In summary, we first identified *GPSM1* as a susceptibility gene for POI, and found it to be associated with follicular development in rats. Then, we confirmed the anti-apoptotic and proliferative functions of GPSM1 in rat GCs, and found that the possible mechanism of action might be through regulation of the Bcl-2/Bax ratio through cAMP-PKA-CREB signaling, which affects the activation of apoptosis protein, Caspase-3, ultimately determining the survival or death of GCs. While further studies are required to verify the results of this study, our findings shed light on the etiology of POI by providing new candidates and pathways to explore.

## Methods

### Ethics statement and patients

This study was approved by and performed in accordance with the Ethics Committee of ZhuJiang Hospital of the Southern Medical University. All patients gave written informed consent for whole-exome sequencing.

A total of 20 unrelated Han Chinese women were recruited. All had at least 6 months of amenorrhea before the age of 40, high FSH plasma levels (> 25 IU/mL) twice at least 4 weeks apart, and a normal 46 XX karyotype. Women having a background of pelvic surgery, anticancer treatment, ovarian infection, autoimmune disease, and/or positive family history were excluded from this study. The clinical characteristics of 20 POI patients are summarized in detail in Supplemental Table 1.

### WES and bioinformatics analysis

Genomic DNA from patients was extracted from peripheral blood leukocytes using standard procedures. WES was performed on patients 1–10 using the SureSelect Human All Exon V6 /V6 + UTR Kit (Agilent Technologies, Santa Clara, CA, USA) on the NextSeq 500 platform from Illumina (San Diego, CA USA). The raw image files were processed into variants with high reliability through several steps of base calling, quality control, alignment, and calibration. Basic sequencing information, including bases, length of reads, depth, and coverage is available in Supplemental Table 2. Single nucleotide variants and indels were then annotated using ANNOVAR. Variants fulfilling the following criteria were retained: (1) absent or rare variants (frequency < 0.01) in the dbSNP, 1000 Genomes Project, and Exome Aggregation Consortium databases; (2) protein structure-altering variants with pathogenicity predicted by SIFT, PolyPhen2, Mutation Assessor, and Mutation Taster.

### Validation of mutation by sanger sequencing

The mutations identified by WES and selected after literature reference were verified by Sanger sequencing in all 20 patients. Primers were designed to amplify the target region flanking the mutation site and are listed in Supplemental Table 3. Purified PCR products were sequenced on an ABI 3730XL (Applied Biosystems, USA) using the BigDye 3.1 Terminator Sequencing Kit (Applied Biosystems, USA) following the manufacturer’s protocol.

### Induction of follicle development and isolation of ovaries in rats

To stimulate follicular growth, immature female Sprague-Dawley (SD) Rats (24–25 days) were intraperitoneally injected with pregnant mare serum gonadotropin (PMSG, 40 IU/rat) (Ningbo Second Hormone Plant, Zhejiang, China). The experimental group (PMSG group, *n* = 3) was treated as above. The control group (NS group, *n* = 3) was intraperitoneally injected with an equal volume of normal saline (NS). Rats were sacrificed 48 h later, and the ovaries were removed immediately and cleaned with phosphate-buffered saline (PBS) for subsequent assays. One ovary of each rat (*n* = 6) was used for RT-PCR, and one for western blotting.

### Immunohistochemistry

Ovaries of mature female SD rats (12 weeks) were removed, formalin fixed, paraffin embedded, sectioned (4 μm), deparaffinized, and rehydrated. Endogenous peroxidase activity was blocked by incubation of the sections with 3% H_2_O_2_ for 15 min. Nonspecific binding was blocked with 5% bovine serum albumin (CWBIO, Jiangsu, China) for 30 min. After washing, sections were incubated overnight at 4 °C with an antibody against GPSM1 (1:200, Proteintech, Wuhan, China), followed by incubation with a biotinylated secondary antibody (1:1000) for 1 h at room temperature. After washing, the antibody complexes were visualized with a DAB Kit (ZSGB-BIO, Shanghai, China) according to the manufacturer’s instructions. The sections were counterstained with hematoxylin, then dehydrated, and mounted.

### Isolation and culture of primary rat GCs

PMSG was intraperitoneally injected into immature female SD rats aged 24–25 days at 40 IU/rat. The rats were sacrificed 48 h later and the ovaries were immediately removed. After washing with PBS, the ovaries were placed in DMEM/F12 medium. GCs were released from the ovarian follicles into the medium by a syringe needle under an anatomic microscope, and then purified by filtration with a 200-μm stainless steel mesh. The isolated GCs were centrifugated at 1000×*g* for 5 min and then resuspended in medium. The GCs were seeded in 6-well plates (1 × 10^6^ cells/well) and cultured in DMEM/F12 containing 1% Penicillin/ Streptomycin and 15% fetal bovine serum at 37 C with 5% CO_2_ for 48 h to allow cells to attach.

### Transfection

Synthetic small-interfering RNAs (siRNAs) were purchased from Sangon Biotech (Shanghai). The sequences of *Gpsm1*-siRNA were 5′-CCUGCGGCACCUUGUCAUUTT-3′, 5′-GCCUAUGGCAACCUGGGUATT-3′, and 5′-CCGAUUCGAUGAGGCAAUUTT-3′, named siRNA-1, siRNA-2, and siRNA-3, respectively. The sequence of siRNA for the negative control was 5′-UUCUCCGAACGUGUCACGUTT-3′, named NC-siRNA. The siRNAs were delivered into the cells with Lipofectamine 3000 (Life Technology, Invitrogen, USA) according to the protocols supplied. The RNA and protein samples were collected 48 h and 72 h after transfection, respectively.

### RNA isolation and RT-PCR

Total RNA was isolated from ovarian tissues or cultured GCs using TRizol reagent (TaKaRa, Japan) according to the manufacturer’s instructions. The first-strand cDNA for total RNA was synthesized using PrimeScript™ RT Reagent Kit with gDNA Eraser (TaKaRa, Japan). The expression levels of mRNA were detected by TB Green Premix Ex Taq II (TaKaRa, Japan) on a Bio-Rad Real-Time PCR system (Bio-Rad Inc., USA). *Gapdh* was used as the internal mRNA control. Target gene expression was determined using the 2^−ΔΔCt^ method. The primer sequences for amplification are listed in Supplemental Table 4.

### Western blot analysis

Total proteins were extracted from ovarian tissues or cultured GCs using RIPA Lysis Buffer (Beyotime Biotechnology, Shanghai, China) containing 1% phosphatase inhibitor and 1% protease inhibitor. The protein samples (15 μg) were loaded onto the sodium dodecyl sulfate polyacrylamide gel for electrophoresis, and then transferred to polyvinylidene difluoride membranes (Merck Millipore, Germany). After blocking in 5% non-fat milk at room temperature for 2 h, primary antibodies against GAPDH (1:500, Proteintech, Wuhan, China), GPSM1 (1:1000, Proteintech, Wuhan, China), PKAc (1:1000, Cell Signal, Beverly, MA, USA), CREB (1:1000, Cell Signal, Beverly, MA, USA), p-CREB (1:1000, Abcam, Cambridge, UK), Bcl-2 (1:1000, AbSci, WA, USA), Bax (1:1000, Proteintech, Wuhan, China), Caspase-3 (1:1000, Cell Signal, Beverly, MA, USA), and cleaved caspase-3 (1:1000, Cell Signal, Beverly, MA, USA) were incubated at 4 °C overnight. The membranes were then incubated with secondary antibodies (1:500, Proteintech, Wuhan, China) for 1 h at room temperature. ECL detection reagent (Merck Millipore, Billerica, MA, USA) was used to visualize the bands. All experiments were repeated at least 3 separate times.

### Cell counting Kit-8 (CCK8) assay

Transfected GCs (2 × 10^3^) were plated into a 96-well plate. At 24, 48,72, 96, and 120 h, cells were treated with 10 μL CCK-8 solution (Corning, Beijing, China) per well. The absorbance value (OD value) of each well was measured at 450 nm on a microplate reader after incubation at 37 °C for 2 h. Each group was established in 5 wells. The proliferation rate was calculated and the cell proliferation curve was drawn. All tests were repeated at least 3 times.

Proliferation rate (%) = (mean OD value at detection time point – OD value in blank group) / (mean 24 h OD value – 24 h OD value in blank group).

### Flow cytometry-based annexin/ propidium iodide (PI) assay

GCs seeded in 6-well plates were transfected with siRNA and cultured for 48 h. Then 1 × 10^6^ cells were collected, washed twice with ice-cold PBS, and resuspended in binding buffer containing Annexin V-FITC and PI. After incubating for 30 min in the dark, cells were analyzed using a BD FACSVerser flow cytometer system (BD Biosciences, USA) equipped with BD FACSuite software.

### Statistical analysis

All data were analyzed using SPSS software (Version 22.0, SPSS Inc., Chicago, USA), and the results are presented as mean ± SEM using at least 3 independent experiments. An unpaired Student t-test was performed when comparing two groups and one-way ANOVA was performed when comparing more than two groups. A *P* value of 0.05 or less was considered statistically significant (*P < 0.05, **P < 0.01).

## Supplementary Information

**Additional file 1:** **Table S1.** Basic clinical characteristics of POI patients. **Table S2.** Raw date of whole exon sequencing. **Table S3.** Primers for Sanger sequencing. **Table S4.** Primers for PCR.

## Data Availability

The raw data of WES required to reproduce these findings cannot be shared at this time as the data also forms part of an ongoing study. Single nucleotide variants and indels were annotated using ANNOVAR (http://www.oponbioinformatics.org/annovar/). The variants were analysed using dbSNP (http://www.ncbi.nlm.nh.gov/snp/), 1000 Genomes Project (1000 g, http://browser.1000genomes.org/index.html), and Exome Aggregation Consortium databases (ExAC, http://exac.broadinstitute.org/).

## Abbreviations

POI: Premature ovarian insufficiency
GPSM1: G protein signaling modulator 1
GCs: Granulosa cells
RT-PCR: Real Time-Polymerase Chain Reaction
CCK8: Cell counting kit 8
siRNA: Small interference RNA
WES: Whole-exome sequencing
FSH: Follicle-stimulating hormone
SNV: Single nucleotide variants
SD: Sprague-Dawley
PMSG: Pregnant mare serum gonadotropin
NS: Normal saline
PBS: Phosphate-buffered saline
AGS3: G protein signaling 3
