# Supplementary material for: Depletion of GPSM1 enhances ovarian granulosa cell apoptosis via cAMP-PKA-CREB pathway in vitro
Source: J Ovarian Res. 2020 Nov 21;13:136. doi: 10.1186/s13048-020-00740-6 (PMC7680585; doi:10.1186/s13048-020-00740-6)
Supplement: Supplementary file 1 — Additional file 1: Table S1. Basic clinical characteristics of POI patients. Table S2. Raw date of whole exon sequencing. Table S3. Primers for Sanger sequencing. Table S4. Primers for PCR. [file 13048_2020_740_MOESM1_ESM.docx]

**Supplemental Table 1.** Basic clinical characteristics of POI patients

| **No.** | **Puberty** | **Age** | **FSH**  **(IU/L)** | **Estradiol**  **(pmol/L)** | **AMH**  **(ng/ml)** | **Karyo**  **type** | **Pelvic ultrasonography** | | |
| --- | --- | --- | --- | --- | --- | --- | --- | --- | --- |
|  |  |  |  |  |  |  | **Ovary size (mm)** | | **Follicle** |
|  |  |  |  |  |  |  | **Right** | **Left** |  |
| 1 | Normal | 26 | 76.03 | 73 | <0.06 | Normal | 25×10×20 | 25×13×22 | Absent |
| 2 | Normal | 36 | 64.27 | 256 | <0.06 | Normal | 27×18×24 | 23×14×21 | Absent |
| 3 | Normal | 34 | 84.76 | 80 | <0.06 | Normal | 24×10×18 | 23×10×17 | Absent |
| 4 | Normal | 20 | 169.06 | 43 | <0.01 | Normal | 19×9×12 | 20×10×14 | Absent |
| 5 | Normal | 28 | 80.4 | 91 | <0.06 | Normal | 24×8×20 | 30×16×25 | Absent |
| 6 | Normal | 28 | 61.01 | 73 | <0.06 | Normal | 31×15×24 | 27×11×21 | Present |
| 7 | Normal | 15 | 58.52 | 90 | <0.06 | Normal | 20×8×19 | 25×11×19 | Absent |
| 8 | Normal | 37 | 43.5 | 38 | 0.02 | Normal | 25×12×17 | 23×12×16 | Absent |
| 9 | Normal | 13 | 102.4 | 48 | <0.01 | Normal | 18×9×10 | 16×10×11 | Absent |
| 10 | Normal | 37 | 83.18 | 173 | <0.06 | Normal | 26×20×23 | 26×20×22 | Absent |
| 11 | Normal | 30 | 81.65 | 73 | <0.06 | Normal | 29×16×27 | 32×18×27 | Present |
| 12 | Normal | 36 | 109.14 | 54 | ＜0.01 | Normal | 22×9×15 | 18×11×14 | Absent |
| 13 | Normal | 37 | 58.55 | 349 | ＜0.01 | Normal | 25×15×23 | 27×15×24 | Present |
| 14 | Normal | 28 | 53.66 | 96 | ＜0.01 | Normal | 13×9×12 | 12×7×11 | Absent |
| 15 | Normal | 32 | 30.2 | 367 | <0.06 | Normal | 20×15×18 | 19×12×13 | Absent |
| 16 | Normal | 29 | 131.07 | 19 | 0.03 | Normal | 25×12×22 | 26×14×24 | Present |
| 17 | Normal | 37 | 26.6 | 125 | <0.06 | Normal | 19×15×13 | 17×10×16 | Present |
| 18 | Normal | 34 | 53.02 | 389 | <0.06 | Normal | 24×16×20 | 29×28×23 | Present |
| 19 | Normal | 39 | 54.4 | 187 | 0.01 | Normal | 24×12×20 | 24×20×37 | Present |
| 20 | Normal | 38 | 63.43 | 67 | 0.01 | Normal | 18×13×10 | 16×12×11 | Absent |

**Supplemental Table 2.** Raw date of whole exon sequencing

| **Patient NO.** | **1** | **2** | **3** | **4** | **5** | **6** | **7** | **8** | **9** | **10** |
| --- | --- | --- | --- | --- | --- | --- | --- | --- | --- | --- |
| Average read length(bp) | 127.5625 | 125.7845 | 128.3623 | 132.0098 | 131.8938 | 111.6757 | 118.066 | 112.2 | 117.2636 | 114.3381 |
| Average sequencing depth near target | 51.482 | 50.585 | 57.572 | 50.729 | 23.242 | 31.796 | 25.997 | 26.491 | 26.508 | 28.985 |
| Average sequencing depth on target | 168.794 | 172.382 | 178.936 | 163.453 | 78.072 | 111.205 | 101.832 | 96.871 | 106.148 | 108.914 |
| Base covered near target | 73136326 | 73211104 | 73321347 | 73211020 | 72470833 | 70699762 | 70686467 | 70692084 | 70309202 | 70938780 |
| Base covered on target | 50311816 | 50318333 | 50308992 | 50306702 | 50296532 | 50301124 | 50303592 | 50298085 | 50301949 | 50301999 |
| Capture Specificity | 0.65581 | 0.67609 | 0.65222 | 0.64668 | 0.6443 | 0.75656 | 0.72976 | 0.73484 | 0.74274 | 0.74345 |
| Coverage of flanking region | 0.98964 | 0.99065 | 0.99214 | 0.99065 | 0.98063 | 0.95667 | 0.95649 | 0.95656 | 0.95138 | 0.9599 |
| Coverage of target region | 0.99844 | 0.99857 | 0.99838 | 0.99834 | 0.99813 | 0.99822 | 0.99827 | 0.99816 | 0.99824 | 0.99824 |
| Duplication Rate | 8.637 | 9.176 | 8.244 | 7.022 | 7.672 | 12.86 | 11.269 | 12.001 | 10.945 | 11.762 |
| Effective bases near target(Mb) | 3804.646 | 3738.309 | 4254.733 | 3749.017 | 1717.641 | 2349.771 | 1921.262 | 1957.721 | 1959.02 | 2142.087 |
| Effective bases on or near target(Mb) | 12310.275 | 12424.757 | 13271.413 | 11985.531 | 5651.732 | 7953.452 | 7052.627 | 6839.098 | 7307.887 | 7630.327 |
| Effective bases on target(Mb) | 8505.628 | 8686.448 | 9016.68 | 8236.514 | 3934.092 | 5603.681 | 5131.364 | 4881.377 | 5348.868 | 5488.24 |
| Fraction of effective bases on or near target | 0.72529 | 0.73916 | 0.72565 | 0.71755 | 0.70599 | 0.79239 | 0.76665 | 0.76873 | 0.77647 | 0.77735 |
| Fraction of effective bases on target | 0.50113 | 0.51676 | 0.49301 | 0.4931 | 0.49143 | 0.55828 | 0.5578 | 0.54867 | 0.56832 | 0.55912 |
| Fraction of flanking region covered with at least 10x | 0.87116 | 0.86056 | 0.90625 | 0.8794 | 0.66613 | 0.62246 | 0.60935 | 0.60595 | 0.60439 | 0.62723 |
| Fraction of flanking region covered with at least 20x | 0.69904 | 0.67945 | 0.75914 | 0.70527 | 0.41764 | 0.45447 | 0.43256 | 0.4305 | 0.43528 | 0.45993 |
| Fraction of flanking region covered with at least 4x | 0.95979 | 0.96023 | 0.97107 | 0.96431 | 0.89271 | 0.80744 | 0.80337 | 0.80069 | 0.79356 | 0.81361 |
| Fraction of target covered with at least 10x | 0.99563 | 0.99626 | 0.99623 | 0.99541 | 0.99051 | 0.9945 | 0.99558 | 0.99466 | 0.99568 | 0.99549 |
| Fraction of target covered with at least 20x | 0.98964 | 0.99159 | 0.99189 | 0.98929 | 0.9709 | 0.98517 | 0.98929 | 0.98634 | 0.98996 | 0.98962 |
| Fraction of target covered with at least 4x | 0.99757 | 0.99783 | 0.99771 | 0.99747 | 0.9964 | 0.99733 | 0.99753 | 0.99729 | 0.99752 | 0.99747 |
| Fraction of uniquely mapped on target | 0.6518 | 0.67075 | 0.6461 | 0.64291 | 0.6406 | 0.74631 | 0.72115 | 0.72352 | 0.7333 | 0.73244 |
| Initial bases near target | 73902222 | 73902222 | 73902222 | 73902222 | 73902222 | 73902222 | 73902222 | 73902222 | 73902222 | 73902222 |
| Initial bases on or near target | 124292823 | 124292823 | 124292823 | 124292823 | 124292823 | 124292823 | 124292823 | 124292823 | 124292823 | 124292823 |
| Initial bases on target | 50390601 | 50390601 | 50390601 | 50390601 | 50390601 | 50390601 | 50390601 | 50390601 | 50390601 | 50390601 |
| Number of reads uniquely mapped to genome | 114148812 | 113026962 | 122776582 | 112138910 | 53877362 | 67228314 | 61634064 | 59569782 | 63024492 | 65747916 |
| Number of reads uniquely mapped to target | 74401763 | 75812976 | 79325768 | 72094695 | 34513848 | 50173040 | 44447436 | 43099811 | 46215868 | 48156107 |
| Reads Mapped To Target Region | 86756909 | 89937988 | 92434400 | 81413612 | 38841322 | 67865730 | 56733048 | 58183090 | 59520060 | 63729429 |
| Reads mapping to genome ratio | 0.99425 | 0.99544 | 0.99468 | 0.99497 | 0.99322 | 0.99804 | 0.99777 | 0.99855 | 0.99844 | 0.9985 |
| Total effective reads | 133055218 | 133635606 | 142480298 | 126532256 | 60695836 | 89879222 | 77916044 | 79292940 | 80261218 | 85849390 |
| Total effective yield(Mb) | 16972.85 | 16809.289 | 18289.102 | 16703.496 | 8005.402 | 10037.321 | 9199.239 | 8896.665 | 9411.716 | 9815.853 |
| Unique reads mapping to genome ratio | 0.85791 | 0.84578 | 0.86171 | 0.88625 | 0.88766 | 0.74799 | 0.79103 | 0.75126 | 0.78524 | 0.76585 |

**Supplemental Table 3.** Primers for Sanger sequencing

| **ID** | **Forward primer（5’-3’）** | **Reverse primer（5’-3’）** |
| --- | --- | --- |
| CNN2 c.629T>C | CTCCACCCCTCCTTCCTCTC | TGCGGGCATAGAAACCACAA |
| CNN2 c.630G>A |  |  |
| CNN2 c.632G>T |  |  |
| CNN2 c.670G>A |  |  |
| CNN2 c.680G>A |  |  |
| CNN2 c.695C>A |  |  |
| PSPH c. 268G>A | ATGCTCTTGGGAGGATGTGC | CGGGGCAGTAAGGCATGTTA |
| SCUBE1 c.1169C>G | ATCAGCCATACCCCAACAGC | GCTTCCAGATGTGGACGAGT |
| GPSM1 c.1840C>A | CTGCGAATCACCCACAGC | GTCCACCCGCTGCTCGTC |

**Supplemental Table 4.** Primers for PCR

| Gene | Primer sequence |
| --- | --- |
| GPSM1 | Forward Prime：5’-GGTACCATGGCAGAGCCACCAGCCTA-3’ |
|  | Reverse Prime：5’-TCTAGATCAGGCTGGCACCCAGCTT-3’ |
| cAMP | Forward Prime：5’-CTCCGTGCTGTGGATGAC -3’ |
|  | Reverse Prime：5’-AAGGCACATTGCTCAGGTAA -3’ |
| PKA | Forward Prime：5’- GCTGGCTTTGATTTACGG -3’ |
|  | Reverse Prime：5’- GATGTTTCGCTTGAGGATA-3’ |
| CREB | Forward Prime：5’- TGAGTTGGCAAGTCCATTCG -3’ |
|  | Reverse Prime：5’- AACGGGCTATCCTGGTGAGT -3’ |
| p-CREB | Forward Prime：5’- AGTGCCCAGCAACCAAGT -3’ |
|  | Reverse Prime：5’- GCTTCCCTGTTCTTCATTAG-3’ |
| Bcl-2 | Forward Prime：5’- CTACGAGTGGGATACTGGAGATGA-3’ |
|  | Reverse Prime：5’- ACAGCCAGGAGAAATCAAACAGA-3’ |
| Bax | Forward Prime：5’- TTGTTACAGGGTTTCATCCAGG-3’ |
|  | Reverse Prime：5’- CAAAGTAGAAGAGGGCAACCAC-3’ |
| Caspase-3 | Forward Prime：5’- TACCAGTGGAGGCCGACTTC-3’ |
|  | Reverse Prime：5’-GCACAAAGCGACTGGATGAAC-3’ |
| GAPDH | Forward Prime：5’-TCCAGGATTGATGACCAGCG-3’ |
|  | Reverse Prime：5’-CACTGGCCTCTTGGTCTGGA-3’ |
